# Development of Personalized Thrombogenesis and Thrombin Generation Assays to Assess Endothelial Dysfunction in Cardiovascular Diseases

**DOI:** 10.3390/biomedicines11061669

**Published:** 2023-06-08

**Authors:** Monica Bacci, Assunta Cancellara, Roberta Ciceri, Erica Romualdi, Valentina Pessi, Fabio Tumminello, Martina Fantuzzi, Marco Paolo Donadini, Corrado Lodigiani, Silvia Della Bella, Francesca Calcaterra, Domenico Mavilio

**Affiliations:** 1 Center for Thrombosis and Hemorrhagic Diseases, IRCCS Humanitas Research Hospital, 20089 Rozzano, Italy; monica.bacci@humanitas.it (M.B.); corrado.lodigiani@humanitas.it (C.L.); 2 Department of Medical Biotechnologies and Translational Medicine, University of Milan, 20089 Rozzano, Italy; assunta.cancellara@unimi.it (A.C.); roberta.ciceri@humanitasresearch.it (R.C.); domenico.mavilio@unimi.it (D.M.); 3 Unit of Clinical and Experimental Immunology, IRCCS Humanitas Research Hospital, 20089 Rozzano, Italy; 4 Centro Trombosi ed Emostasi, Ospedale di Circolo e Fondazione Macchi, ASST Sette Laghi, 21100 Varese, Italy; erica.romualdi@asst-settelaghi.it (E.R.); marcopaolo.donadini@asst-settelaghi.it (M.P.D.); 5 UO Medicina 2, Ospedale di Circolo e Fondazione Macchi, ASST Sette Laghi, 21100 Varese, Italy; 6 Dipartimento di Medicina e Chirurgia, Università Dell’Insubria, 21100 Varese, Italy; 7 Department of Biomedical Sciences, Humanitas University, Via Rita Levi Montalcini 4, 20090 Pieve Emanuele, Italy

**Keywords:** endothelial dysfunction, endothelial colony-forming cells (ECFCs), thrombogenesis assay, thrombin generation assay (TGA)

## Abstract

The study of endothelial dysfunction (ED) is crucial to identify the pathogenetic mechanism(s) and provide indications for patient management in cardiovascular diseases. It is currently hindered by the limited availability of patient-specific primary endothelial cells (ECs). Endothelial colony-forming cells (ECFCs) represent an optimal non-invasive tool to overcome this issue. Therefore, we investigated the use of ECFCs as a substrate in thrombogenesis and thrombin generation assay (TGA) to assess ED. Both assays were set up on human umbilical vein endothelial cells (HUVECs) and then tested on ECFCs obtained from healthy donors. To prove the ability of the assays to detect endothelial activation, ECs stimulated with TNFα were compared with unstimulated ECs. EC activation was confirmed by the upregulation of VCAM-1 and Tissue Factor expression. Both assays discriminated between unstimulated and activated HUVECs and ECFCs, as significantly higher platelet deposition and fibrin formation in thrombogenesis assay, and thrombin generation in TGA, were observed when TNFα-activated ECs were used as a substrate. The amount of fibrin and thrombin measured in the two assays were directly correlated. Our results support the combined use of a thrombogenesis assay and TGA performed on patient-derived ECFCs to provide a personalized global assessment of ED relevant to the patient’s hemostatic profile.

## 1. Introduction

The vascular endothelium consists in a single layer of endothelial cells (ECs) that lines the interior surface of all blood vessels being in direct contact with blood soluble and cellular components. It regulates exchanges between the bloodstream and the surrounding tissues and plays a critical role in cardiovascular homeostasis [1,2]. In particular, ECs are involved in regulating blood fluidity, vascular tone, angiogenesis, monocyte/leukocyte adhesion, platelet aggregation, and activation of the coagulation and fibrinolytic cascade [1,2]. As a consequence, it has been widely recognized that endothelial dysfunction (ED) is deeply involved in the pathogenesis of cardiovascular disorders [3]. ED is characterized by impaired endothelium-dependent vasodilatation, redox balance, and increased inflammatory reactions within the blood vessel wall [4]. In addition, another feature of ED is EC activation with upregulation of cytokines, adhesion molecules, and other molecules involved in cell–cell interactions, thus leading to a prothrombotic and pro-inflammatory state in the blood vessels [5,6]. Actually, increasing evidence supports the crucial role of inflammation in driving the EC activation/dysfunction involved in the pathogenesis of cardiovascular diseases such as hypertension, atherosclerosis, stroke, ischemic heart disease, venous thrombosis, and intimal hyperplasia [2,7]. In fact, several risk factors for cardiovascular diseases (i.e., poor diet, smoking, diabetes, obesity, and physical inactivity) are conditions that contribute to a pro-inflammatory state [8].

In order to shed light on the molecular mechanisms responsible for ED and their role in the pathogenesis of distinct cardiovascular disorders, in vitro models that allow characterizing ED by modeling patient-specific endothelial function are required. However, a patient-specific approach is hindered by the limited availability of patient-derived primary ECs. In this scenario, endothelial colony-forming cells (ECFCs) represent an optimal tool for studying ED. ECFCs can be isolated and expanded from the peripheral blood, are endowed with typical endothelial morphology, express endothelial but not hematopoietic markers, and exhibit clonal proliferative potential [9,10,11,12]. Besides representing a promising cell source for the revascularization of damaged tissues [13], ECFCs can be used as a liquid biopsy that gives access to patient ECs in a non-invasive way, thus allowing to study of ED in distinct diseases [14,15].

The thrombogenesis assay and the thrombin generation assay (TGA) are commonly used to assess the coagulation and thrombotic profile of patients with bleeding or thrombotic disorders. In particular, the thrombogenesis assay is used to investigate the prothrombotic/antithrombotic state of patients, and it is performed by in vitro flow-based methods that allow studying the role of platelets and coagulation in hemostasis and thrombosis. The most broadly used thrombogenesis assay involves perfusing whole blood over immobilized fibrillar collagen and measuring platelet adhesion and fibrin formation [16]. TGA is a global dynamic assay developed to measure a patient’s hemostatic balance [17]. It is considered superior to routine coagulation tests in measuring the hemostatic function because it records the final products of the coagulation cascade, whereas prothrombin time and activated partial thromboplastin time only evaluate the time to the start of clot formation [18]. Among other approaches, thrombin generation via calibrated automated thrombogram (CAT) is widely used as a potential global coagulation marker for predicting thrombotic risk [18]. Originally, it was developed to monitor thrombin formation in plasma samples upon activation of the coagulation cascade by soluble tissue factor [19].

Variants of both assays have been developed in cultured ECs to explore mechanisms of prothrombotic states in diseases with endothelial dysfunction, allowing an overall assessment of coagulation activators and inhibitors [20].

Based on the advantages of using patient-derived ECFCs to study ED in vascular diseases, in this study, we set up the experimental conditions to perform the thrombogenesis assay and the TGA modified using ECFCs as a substrate. Both the experimental settings were first optimized in a few experiments performed on human umbilical vein endothelial cells (HUVECs), which are the most popular model used for studying ECs in vitro. Both assays were then applied to ECFCs isolated from healthy donors. To assess whether the assays allowed a proper assessment of endothelial alterations, HUVECs, and ECFCs were used in the assays either in basal conditions or after in vitro activation with TNFα.

Both assays demonstrated the ability to discriminate between unstimulated and activated cells on HUVECs and ECFCs, thus supporting the use of ECFCs-modified thrombogenic assay and TGA for patient-specific investigation of ED and paving the way for the application of these assays in personalized modeling of vascular diseases.

## 2. Materials and Methods

### 2.1. Human Blood Samples

Blood samples were obtained by venipuncture from healthy volunteers with no history of thrombosis or bleeding and who had not taken anti-inflammatory drugs for at least 10 days. In particular, samples dedicated to ECFC isolation were collected into heparin Vacutainer tubes (BD Biosciences, San Jose, CA, USA), whereas samples perfused in thrombogenesis assay and used for platelet-rich plasma (PRP) preparation were collected in vacutainer tubes containing 0.109 M trisodium citrate as anti-coagulant (BD Biosciences, San Jose, CA, USA). All samples were processed and analyzed within 3 h after collection.

### 2.2. Cell Preparation

#### 2.2.1. ECFC Isolation and Expansion

ECFCs were isolated and cultured from peripheral blood mononuclear cells (PBMCs) obtained from 9 healthy donors, according to methods previously described [15,21,22,23]. Briefly, PBMCs isolated from 50 mL of heparinized blood using a density-gradient centrifugation were further resuspended in EGM-2 medium (Lonza Inc., Allendale, NJ, USA) + 5% Fetal Bovine Serum (FBS, Euroclone, Milan, Italy) and seeded at a final density of 2.5 × 10^6^ cells/cm^2^ onto 24-well tissue culture plates precoated with human fibronectin (Sigma-Aldrich, St. Louis, MO, USA) diluted in Phosphate Buffered Saline (PBS, Euroclone, Milan, Italy) to the final concentration of 1 μg/cm^2^. After one day of culture, the EGM-2 medium was removed, thus allowing the removal of debris and nonadherent cells. The remaining adherent cells were then washed with EGM-2 medium, and finally, fresh EGM-2 medium was added to each well. The medium was changed every 2 days until the colony appeared as was monitored by daily inspection using an inverted microscope. ECFC colonies were detached with Trypsin-EDTA 1x (Euroclone, Milan, Italy) from the original tissue culture plates and subpassaged in flasks precoated with type I collagen (Corning Inc., Corning, NY, USA) diluted in acetic acid 0.02 N to the final concentration of 50 µg/mL. The ECFC nature of the isolated colonies was assessed, as previously described [15,21,22], based on their typical cobblestone-like morphology, their high proliferative potential, and their random checked endothelial immunophenotype, characterized by the expression of the endothelial markers CD31, CD34, VEGFR2, and CD146 and lack of the hematopoietic markers CD45 and CD14. Cell count and viability were assessed at each passage using a hemacytometer and trypan blue exclusion. ECFCs in passages 4–6 were used in the described experiments.

#### 2.2.2. Human Umbilical Vein Endothelial Cell (HUVEC) Expansion

Commercially available HUVECs (Merck-Millipore, Billerica, MA, USA) were seeded at a density of 10.000 cells/cm^2^ in flasks precoated with type-I collagen (50 µg/mL), cultured in EGM-2 + 5% FBS. HUVECs in passages 4–6 were used in the described experiments.

#### 2.2.3. Endothelial Cells (ECs) In Vitro Activation

For EC in vitro activation, both HUVECs and ECFCs cultured in EGM-2 + 5% FBS were incubated with TNFα (20 ng/mL; PeproTech, London, UK) for 4 h at 37 °C.

### 2.3. Flow Cytometry

#### 2.3.1. Assessment of ECFC Immunophenotype

The immunophenotype of ECFC colonies was confirmed by assessing by flow cytometry the expression of endothelial markers (CD31, CD34, VEGFR-2, and CD146) and the absence of the hematopoietic markers CD45 and CD14 [15,21,22]. Briefly, ECFCs were detached with Accutase Cell Detachment Solution 1× (Millipore, Billerica, MA, USA) and stained with Zombie Aqua dye (BioLegend, San Diego, CA, USA) for 15 min at room temperature (RT) to discriminate viable cells. Cells were then washed and incubated for 20 min at RT with BV480-conjugated anti-human CD31 (Clone: M89D3; BD PharMingen, San Diego, CA, USA), PE Cy7-conjugated anti-human CD34 (Clone: 581; BD PharMingen, San Diego, CA, USA), PE-conjugated anti-human VEGFR-2 (Clone: 89106; R&D, Minneapolis, MN, USA), BV421-conjugated anti-human CD146 (Clone: P1H12; Biolegend, San Diego, CA, USA), APC Cy7-conjugated anti-human CD45 (Clone: HI30; Biolegend, San Diego, CA, USA), and BV570-conjugated anti-human CD14 (Clone: M5E2; Biolegend, San Diego, CA, USA). Staining conditions for each mAb were preliminarily determined in titration assays. After staining, cells were fixed with 1% paraformaldehyde (PFA, Sigma-Aldrich, Saint Louis, MO, USA) and then acquired within 24 h using a BD LSR Fortessa flow cytometer (BD Biosciences, San Jose, CA, USA). FACS data were analyzed with FlowJo software version 9.9.6 (BD Biosciences, San Jose, CA, USA) and were compensated by using single-stained antibody-capture beads (CompBeads, BD Biosciences, Heidelberg, Germany). Fluorescence Minus One (FMO) controls for each of these molecules were performed. Cells were electronically gated according to light scatter properties to exclude cell debris. Data were expressed as a frequency of positive cells gated on viable cells.

#### 2.3.2. Assessment of EC Activation in Response to TNFα Treatment

EC activation in response to TNFα-treatment was confirmed by assessing the expression of the adhesion molecule Vascular Cell Adhesion Molecule-1 (VCAM-1 or CD106) and the pro-coagulant molecule Tissue Factor (TF) by flow cytometry. Briefly, HUVECs and ECFCs, cultured either in basal conditions or after incubation with TNFα, were detached with Accutase Cell Detachment Solution 1× (Merck-Millipore, Billerica, MA, USA) and stained with Zombie Aqua dye (BioLegend, San Diego, CA, USA) for 15 min at RT to discriminate viable cells. Subsequently, cells were washed and then incubated for 20 min at RT with FITC-conjugated anti-human VCAM-1 (Clone: 1.G11B1; Bio-Rad AbD Serotec, Kidlington, UK), and PE-conjugated anti-human TF (Clone: HTF-1; BD PharMingen, San Diego, CA, USA) monoclonal antibodies (mAbs). Staining conditions for each mAb were preliminarily determined in titration assays. After staining, cells were fixed with 1% paraformaldehyde (PFA, Sigma-Aldrich, Saint Louis, MO, USA) and then acquired within 24 h using the FACSCanto II flow cytometer (BD Biosciences, San Jose, CA, USA). FACS data were analyzed with FlowJo software version 9.9.6 (BD Biosciences, San Jose, CA, USA) and were compensated by using single-stained antibody-capture beads (CompBeads, BD Biosciences, Heidelberg, Germany). FMO controls for each of these molecules were performed. Cells were electronically gated according to light scatter properties to exclude cell debris. Data were expressed as mean fluorescence intensity (MFI) and as the frequency of positive cells gated on viable cells.

### 2.4. Thrombogenesis Assay

#### 2.4.1. EC Preparation and Chamber Characteristics

ECs, either HUVECs or ECFCs, were seeded on a glass-coverslip (24 × 50 mm, Deckglaser Fisher Scientific, Schwerte, Germany) coated with type I collagen (50 µg/mL; Corning Inc, Corning, NY, USA) at a cell density of 10.000 cells/cm^2^ and were cultured in EGM-2 + 5% FBS. Once ECs were confluent, the coverslip with seeded cells was assembled in the flow chamber. The flow chamber had a flow channel defined by a silicon layer of 3 cm length, 0.3 cm width, and 0.0125 cm height, in which perfused whole blood carried the cells through an inlet and an outlet [24,25,26]. Before proceeding with perfusion, ECs were stained with AF647-conjugated anti-human CD31 mAb (Clone: M89D3; BD PharMingen, San Diego, CA, USA) diluted 1:100 in HEPES buffer (20 mM Hepes, 135 mM NaCl, pH 7.4) addition with 5% FBS in order to assess the plastic adhesion and confluence of ECs before and after perfusion. 

#### 2.4.2. Preparation of Whole Blood Samples for Perfusion

Blood samples collected from healthy donors were perfused in the thrombogenesis assay. Before perfusion, 500 µL of whole blood were incubated with 5 µL of FITC-conjugated mouse anti-human GpIbα mAb (CD42b, clone: SZ2; Beckman Coulter, Krefeld, Germany), and 1 µg/mL of R-PE-conjugated mouse anti-human fibrin mAb (Clone: 64C5; MERU VasImmune, San Diego, CA, USA) for 10 min. The anti-GpIbα antibody specifically binds the alpha chain of GpIb on platelets, thus allowing the detection of platelet aggregates. The used anti-fibrin antibody targets a human fibrin neo-epitope only during fibrinogen cleavage by thrombin, thus allowing a precise and specific evaluation of fibrin formation [27]. Stained whole blood was then reconstituted with 10 mM CaCl_2_ immediately before perfusion to reconstitute citrated anticoagulated blood.

#### 2.4.3. Flow Chamber Perfusion

The previously described chamber was mounted on an inverted confocal microscope (TCS Sp5, Leica Microsystems, Wetzlar, Germany) to detect platelet deposition and fibrin formation. ECs were perfused with stained whole blood in the flow chamber for 5 min at a regular rate of 300 s^−1^ at 37 °C by using a syringe pump (New Era Pump System Inc., Farmingdale, NY, USA), thus mimicking a physiologic shear rate occurring in veins [28,29]. Immediately after EC perfusion, platelet, and fibrin deposition, supported by endothelial activation and TF exposure, was assessed by Z-stacks acquisition. All experiments were conducted maintaining constant microscope settings, including laser intensity, voltage, magnification, and pinhole aperture [30]. For each sample, Z-stacks were acquired in three different areas along the frame.

#### 2.4.4. Quantification of Platelet Aggregates and Fibrin Deposition

ImageJ Software version 1.52t (NIH, Bethesda, MD, USA) was used to quantify platelet deposition and fibrin formation. Briefly, 512 × 512-pixel images were taken with a 40× magnification, and Z-stacks were acquired with 0.97 µm step size. Images were acquired in LIF format and then converted into binary images of 8-bit grayscale. For analyses of segmentation, specific thresholds were set for GpIb and fibrin signals in order to exclude the background [31]. Images collected from the same samples before perfusion were used as negative controls [31]. By Z-stack analyses, we estimated the volume (µm^3^) occupied by platelet aggregates and fibrin. Platelet deposition and the fibrin formation occurring only on the endothelial layer were considered.

### 2.5. Thrombin Generation Assay (TGA)

#### 2.5.1. Platelet-Rich Plasma (PRP) Preparation

In order to obtain PRP, whole blood samples were centrifuged (150× *g* for 15 min, without a break). Platelet count in PRP was estimated with a hematology analyzer DXH 800 (Beckman Coulter, Krefeld, Germany). An aliquot of PRP was further centrifuged (2500× *g* for 10 min) in order to obtain platelet-poor plasma (PPP) that was used to adjust platelet count in PRP at the original platelet concentration before using PRP samples in TGA on ECs.

#### 2.5.2. TGA on ECs

We modified TGA, as originally described by Hemker and colleagues [32], by using ECs as triggers for thrombin formation. In particular, ECs—either commercially available HUVECs or ECFCs obtained from healthy controls—were cultured in basal conditions or stimulated with TNFα and used as a substrate in the assay. Confluent ECs were detached with Accutase Cell Detachment Solution (Merck-Millipore, Billerica, MA, USA), and their concentration was then adjusted with Dulbecco’s Phosphate Buffered Saline (DPBS; Sigma-Aldrich, Saint Louis, MO, USA) in order to use 20.000 ECs/well in a 96-well-plate. This optimal cell concentration was determined in preliminary experiments performed on both HUVECs and ECFCs. In each well, ECs resuspended in 20 µL of PBS were mixed with 80 µL of PRP, and thrombin generation was measured with a Calibrated Automated Thrombography and Fluoroskan Ascent Fluorometer (Thermo Scientific, Vantaa, Finland) after the addition of the fluorogenic substrate containing CaCl_2_ (Fluo kit, Diagnostica Stagò, Gennevilliers, France). Each condition tested was associated with a Thrombin Calibrator in which thrombin concentration was known (Thrombin Calibrator, Diagnostica Stagò, Gennevilliers, France). After 1 h of reaction, the TGA parameters Lag Time (minute, min), Peak (nM), and Endogenous Thrombin Potential (ETP) (nM·min) were calculated by Thrombinoscope software (version 5.0, Thrombinoscope BV, The Netherlands).

### 2.6. Statistical Analysis

The Wilcoxon matched-pairs signed rank test was used for comparisons between samples. The Spearman rank correlation test was used to analyze the relationship between variables. All statistical analyses assumed a two-sided significance level of 0.05. Data analyses were performed with Openstat software (version 11.9.08; Softonic International, Barcelona, Spain).

## 3. Results

### 3.1. ECFCs Are Responsive to TNFα Stimulation

In the experiments of thrombogenesis and TGA performed in this study, the activated ECs were obtained, incubating cells with TNFα, a pro-inflammatory cytokine known to promote ED. In order to confirm the activation state of HUVECs and ECFCs upon TNFα treatment, the surface expression levels of the adhesion molecule VCAM-1 and the pro-coagulant molecule TF were assessed by flow cytometry. In particular, VCAM-1 was chosen as one of the most commonly used markers of endothelial activation [33,34], whereas TF was chosen because of its direct involvement in thrombin generation [35].

**Figure 1:**
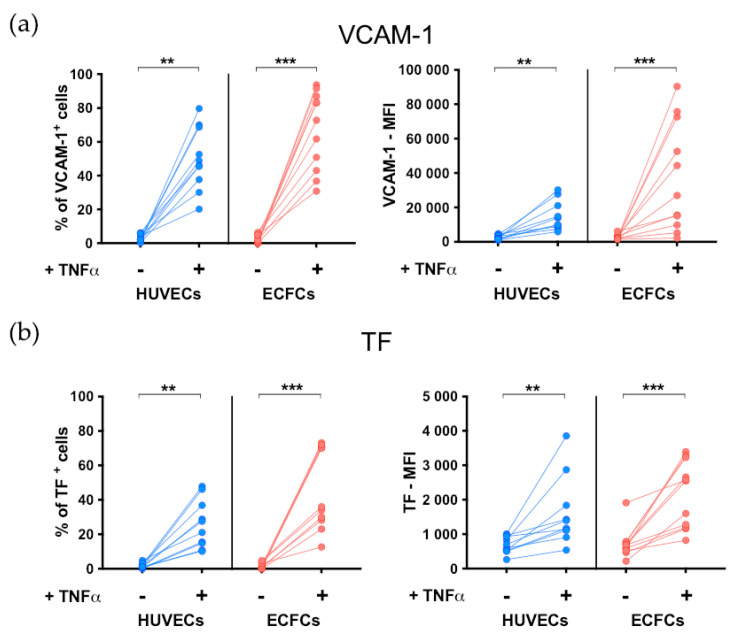
Evaluation of EC activation in response to TNFα treatment. The effect of TNFα stimulation on the activation state of HUVECs (n = 10) and ECFCs (n = 11) was confirmed by flow cytometry evaluating the expression of (**a**) the adhesion molecule VCAM-1 and (**b**) the pro-coagulant molecule Tissue Factor (TF). Data are expressed as a frequency of positive cells (left) and as mean fluorescence intensity (MFI) (right). ** *p* < 0.01 and *** *p* < 0.001 are treated ECs vs. untreated ECs. Statistical significance was calculated using the Wilcoxon matched-pairs signed rank test.

As expected, in basal conditions HUVECs did not express VCAM-1 and TF, but their expression was significantly increased by TNFα treatment (both *p* < 0.01; Figure 1a,b). Similar to HUVECs, ECFCs also did not express VCAM-1 and TF in basal conditions but significantly increased the surface expression of both molecules upon TNFα stimulation (both *p* < 0.001; Figure 1a,b). In all experiments, the TNFα-induced upregulation of VCAM-1 and TF was observed both as an increased frequency of positive cells and as increased MFI.

### 3.2. ECFCs Are a Suitable Substrate for the Thrombogenesis Assay

In order to assess whether ECFCs could be used as a substrate in the thrombogenesis assay, we first set up the method on HUVECs, and then we tested the assay on ECFCs. To verify whether the assay allowed to detect endothelial activation, both the HUVECs and ECFCs were used as substrates in the assay either in basal conditions or after in vitro stimulation with TNFα.

**Figure 2:**
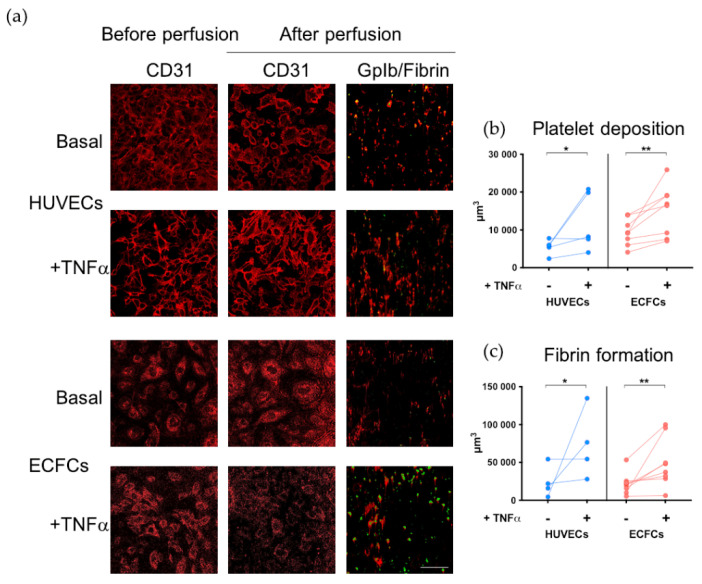
Validation of ECFCs as a substrate for thrombogenesis assay. Evaluation by confocal microscopy of platelet deposition and fibrin formation on the endothelial layer under flow conditions. (**a**) Representative images of HUVECs and ECFCs seeded on glass coverslips before (first column) and after perfusion (second column). Cell confluence was assessed by CD31 staining (in red). Third column: representative images showing platelet deposition (assessed by GpIb staining, in green) and fibrin formation (assessed by fibrin staining, in red) on layers of HUVECs and ECFCs cultured in basal condition and after incubation with TNFα. Images were acquired with confocal microscopy (Leica TCS SP5; magnification 40×). Scale bar: 100 μm. (**b**) Platelet deposition was assessed on HUVECs (n = 5) and ECFCs (n = 8) cultured in basal conditions and after incubation with TNFα. (**c**) Fibrin formation was assessed on HUVECs (n = 4) and ECFCs (n = 8) cultured in basal conditions and after incubation with TNFα. Data are expressed as volume (μm^3^). * *p* < 0.05 and ** *p* < 0.01 treated ECs vs. untreated ECs. Statistical significance was calculated using the Wilcoxon matched-pairs signed rank test.

First, we verified that the platelet deposition and the fibrin formation measured in the thrombogenesis assay occurred only on the endothelial layer and not on the exposed collagen used as a substrate for cell adhesion to the coverslips. To this aim, cell confluence was verified by confocal microscopy prior to and after blood perfusion by identifying ECs on the basis of CD31 expression. As shown in Figure 2a, the layer of HUVECs remained intact after blood perfusion and similar results were obtained when ECFCs were used as a substrate. Then, we quantified platelet deposition and fibrin formation to assess whether our experimental conditions allowed us to distinguish between quiescent and activated ECs. As shown in Figure 2b, platelet deposition was significantly increased when blood was perfused on TNFα-treated HUVECs compared with their unstimulated counterpart (*p* < 0.05). Similarly, also fibrin formation was significantly increased on TNFα-activated compared with unstimulated HUVECs (*p* < 0.05; Figure 2c).

Similar results were confirmed when ECFCs instead of HUVECs were used as a substrate. Indeed, both platelet deposition (*p* < 0.01; Figure 2b) and fibrin formation (*p* < 0.01; Figure 2c) were significantly increased when TNFα-treated ECFCs were used as substrate compared to ECFCs cultured in basal conditions.

Altogether, these results indicate that ECFCs are a suitable substrate for the thrombogenesis assays and that the experimental conditions optimized in our setting allow the detection of EC activation not only on HUVECs but also when ECFCs are used.

### 3.3. ECFCs Are a Suitable Substrate for TGA

In order to assess whether ECFCs could be used as a substrate in an EC-adapted TGA, similar to the approach we used for the setting of the thrombogenesis assay, we first set up the method on HUVECs, and then we tested the assay on ECFCs obtained from healthy donors. HUVECs and ECFCs cultured in either basal condition and upon TNFα stimulation were used to assess whether the assay allowed the distinction between basal and activated ECs.

**Figure 3:**
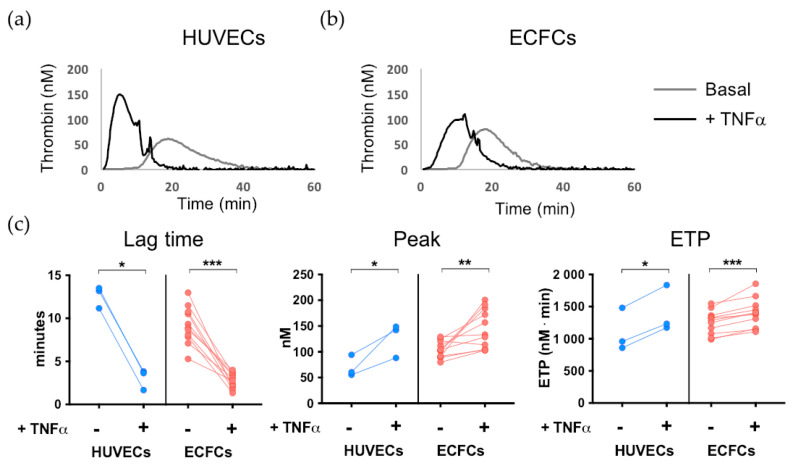
Validation of ECFCs as substrate for TGA on ECs. (**a**,**b**) Representative thrombogram of TGA performed using (**a**) HUVECs and (**b**) ECFCs as a substrate. In both plots, the following conditions are shown: PRP + untreated ECs (basal, gray line), PRP + TNFα-activated ECs (+TNFα, black line). (**c**) Thrombin generation was assessed by TGA on HUVECs (n = 3) and ECFCs (n = 11) cultured in basal conditions and after incubation with TNFα. The following parameters were assessed: Lag time (min), thrombin formation peak (nM), and endogenous thrombin potential (ETP) (nM⋅min). * *p* < 0.05; ** *p* < 0.01; and *** *p* < 0.001 treated ECs vs. untreated ECs. Statistical significance was calculated using the Wilcoxon matched-pairs signed rank test.

As shown in the representative thrombogram reported in Figure 3a, thrombin generation occured faster and was significantly increased when TNFα-treated HUVECs were used as a source of TF compared to HUVECs cultured in basal conditions. In particular, as shown in Figure 3b, the Lag Time was significantly reduced (*p* < 0.05), whereas the amount of thrombin production assessed either as Peak and as ETP was significantly increased (both *p* < 0.05) when TNFα-treated compared with unstimulated HUVECs were used as a substrate.

Similar results were obtained when ECFCs, instead of HUVECs, were used as a substrate in TGA. As shown in the representative thrombogram reported in Figure 3a, thrombin generation occured faster and was significantly increased when activated TNFα-treated ECFCs were used as substrate in the assay compared with ECFCs cultured in basal conditions. Accordingly, the Lag Time was significantly reduced (*p* < 0.001), and the amount of thrombin production assessed either as Peak and as ETP was significantly increased (Peak *p* < 0.01; ETP *p* < 0.001) when TNFα-treated ECFCs were used as a substrate compared with their unstimulated counterpart (Figure 3b). These results indicate that ECFCs are a suitable substrate also for the TGA and that the experimental setting optimized in this study allows the distinction between basal and activated cells, thus enabling the detection of dysfunctional ECs.

**Figure 4:**
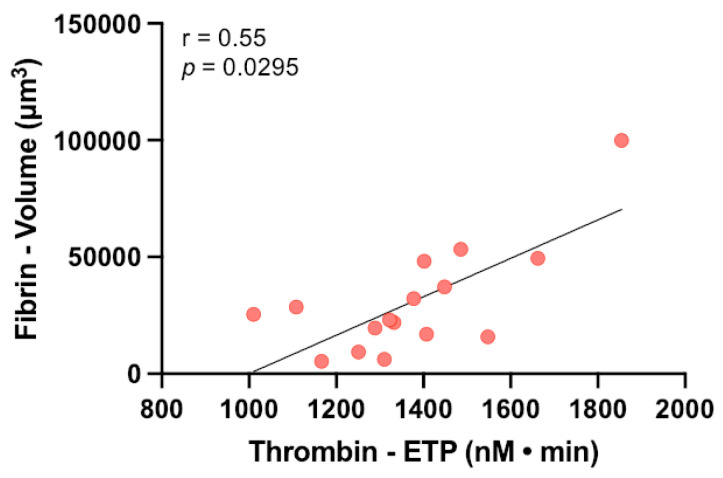
Correlation analysis between fibrin formation occurring in thrombogenesis assay and thrombin generation measured in TGA. The correlation between the volume of fibrin formed on ECFC layers in thrombogenesis assay and the total amount of thrombin generated in TGA on ECFCs expressed as ETP was evaluated. Data from 8 ECFC colonies cultured either in basal conditions or after TNFα activation were used to assess the correlations. Correlation between the assessed parameters was evaluated with Spearman correlation analysis.

Finally, because thrombin is an enzyme that converts fibrinogen into fibrin, we investigated whether the amount of thrombin generated in TGA on ECFCs and the amount of fibrin formed in the thrombogenesis assay performed on the same ECFC layers were correlated. To this aim, we considered the results obtained by performing the two assays on 8 different ECFC colonies analyzed either in basal conditions or after TNFα treatment. As shown in Figure 4, the amount of fibrin measured in the thrombogenesis assay directly correlated with the amount of thrombin generated in TGA, either assessed as Peak or ETP (a correlation between fibrin volume and Thrombin Peak: *r* = 0.3912, *p* = 0.1351; a correlation between fibrin volume and Thrombin ETP: *r* = 0.5500, *p* < 0.05), thus suggesting that the combined use of these assays may provide a global assessment of ED, and may be useful to investigate the ability of ECFCs to promote thrombus formation in patient-specific settings.

## 4. Discussion

ECs have long been studied for their involvement in various physiopathologic processes, from angiogenesis to inflammation and vasoregulation. Moreover, ECs play a key role in hemostasis switching from an anti-coagulant to a pro-coagulant profile following exposure to shear stress and upregulation of surface molecules as a consequence of their activated state [3]. Considering the EC role in the regulation of these processes, it is not surprising that functional alterations of the endothelial compartment are deeply implicated in the pathogenesis of several diseases [3]. For these reasons, there is a growing interest in the study of ED, as ECs are emerging as an important therapeutic target in predictive, preventive, and personalized (3P) medicine with utmost importance in vascular diseases [33].

Several strategies have been developed to study ED in cardiovascular diseases, ranging from non-invasive assessment of endothelial function with flow-mediated dilatation (FMD) of the brachial artery to measurement of peripheral blood biomarkers of ED—such as pro-inflammatory molecules, soluble adhesion molecules, and molecules that participate in the coagulation pathway such as von Willebrand factor (vWF) and thrombomodulin—as they represent a simple and non-invasive method for assessing the integrity of endothelial function [33]. In addition, cellular biomarkers of ED, such as microparticles, circulating endothelial cells (CECs), and circulating endothelial progenitor cells (EPCs), are used as strategies to evaluate ED and the balance between vascular endothelial damage and repair [33,36]. However, all these strategies give indications about the presence of ED by providing indirect information on endothelial function. In order to investigate the mechanism(s) underlying ED by directly addressing ECs, there is increasing interest in the use of ECFCs as an optimal non-invasive approach to studying patient-specific endothelium [9,14,15]. Therefore, in this study, we aimed to set up two functional assays commonly used to assess the coagulation and thrombotic profile of patients with bleeding or thrombotic disorders, properly modified to assess the contribution of ED in the whole process. In particular, we modified the TGA and the thrombogenesis assay to investigate the ability of ECFCs to promote thrombosis by monitoring their contribution to the different phases of thrombus formation. In the thrombogenesis assay performed on ECFC layers, we evaluated the platelet adhesion—the first phase of hemostasis—and the fibrin formation—the last phase of hemostasis. In the TGA performed on ECFCs, we assessed the formation of thrombin that, in turn, is involved in fibrin formation. Both assays were first optimized on HUVECs—primary endothelial cells commonly used in in vitro models of vascular research—and then tested on ECFCs obtained from healthy donors. In addition, as TNFα is the most typical pro-inflammatory stimulus able to activate ECs [37,38,39], TNFα-treated HUVECs and ECFCs were compared with their untreated counterparts to verify that both assays were able to discriminate between activated and non-activated cells, thus proving their ability to detect inflammation-induced ED. We preliminary confirmed HUVEC and ECFC activation in response to TNFα treatment by demonstrating an increased expression of VCAM-1 and TF that are typically expressed by activated ECs [33,40,41]. In particular, VCAM-1 is involved in the adhesion of leukocytes to ECs, whereas TF is a plasma membrane-anchored glycoprotein that acts as the primary initiator of the coagulation cascade [33,40,41].

By comparing HUVECs cultured in basal conditions with cells activated in vitro with TNFα, we confirmed that the two functional assays developed in this study allowed the assessment of the activation status of ECs. In fact, increased platelet deposition and fibrin formation were observed in the thrombogenesis assay as well as increased and faster thrombin generation in the TGA when TNFα-treated HUVECs were used as substrate compared with their untreated counterparts. Importantly, similar results were obtained when ECFCs, instead of HUVECs, were used as a substrate in the assays.

Our results thus confirmed that ECFCs could be successfully used as a substrate in the thrombogenesis assay and in TGA. This observation is in accordance with previous studies that used ECFCs in functional assays in different settings [20,42,43,44]. In this study, by demonstrating the ability of the assays to detect changes in the behavior of TNFα-stimulated compared with unstimulated ECFCs, we further proved that ECFC-modified thrombogenesis assay and TGA could be used to profile thrombotic and coagulation properties that are a direct consequence of endothelial activation. Notably, the shear rate used in the thrombogenesis assay in this study was chosen to mimic a rate occurring in the venous compartment [28,29] that may be relevant when addressing endothelial dysfunction in venous thrombosis. As the shear rate differs in different arterial and venous districts throughout the body, this parameter can be modified according to the properties of the vascular bed of interest [28,29,45].

Moreover, by performing the two functional assays using the same ECFC colonies, we demonstrated a significant positive correlation between the amount of fibrin formed in the thrombogenesis assay and the amount of thrombin generated in TGA. Because both fibrin and thrombin in the ECFC-modified assays are induced by the ECs used as a substrate, this observation confirms the reliability of the developed assays in assessing ECFC function. Moreover, because thrombin, which is measured in the TGA, is directly involved in the generation of fibrin, which is measured in the thrombogenesis assay, we suggest that the combined use of ECFC-modified assays can provide an unprecedented global dynamic assessment of endothelial function. In this respect, the superiority of TGA over routine coagulation assays in assessing the hemostatic balance is widely accepted [17]. In fact, TGA is a global assay allowing the dynamic continuous, and simultaneous recording of the combined effects of both thrombin generation and thrombin inactivation. In its original version proposed by Hemker [32,44,46], the amount of generated thrombin relied on a fixed amount of exogenous TF. In the ECFC-modified TGA developed in this study, the amount of generated thrombin reflects the whole activation status of the tested ECs, thus providing a measure of the balance between the ECFC expression of pro-coagulant factors, including TF, and anti-coagulant factors. Despite the cost and time-consuming issues and limits deriving from possible low ECFC yield in patients characterized by ED, the combined use of the assays is worthwhile. In fact, by combining ECFC-modified TGA with ECFC-modified thrombogenesis assay, we increase the complexity of endothelial function assessment, providing global information on the impact of ECFCs on the entire hemostatic process, from platelet adhesion to fibrin formation.

Applied to patients with cardiovascular diseases, the combined use of thrombogenesis assay and TGA performed on patient-derived ECFCs has great potential in providing a personalized global assessment of ED.

## Data Availability

Data will be made available on request.
